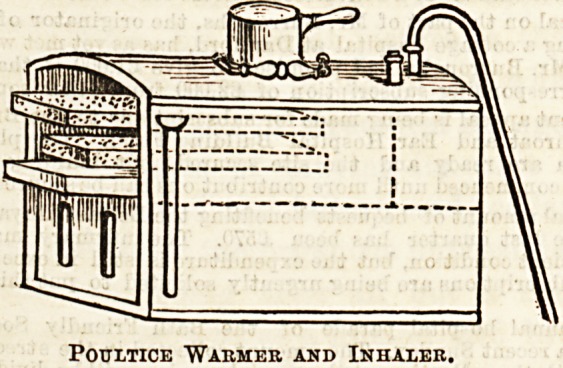# Poultice Warmer and Inhaler

**Published:** 1893-10-07

**Authors:** 


### PRACTICAL DEPARTMENTS.

### POULTICE WARMER AND INHALER.

This invention, for which provisional protection has been obtained, strikes us as one of the most useful of those simple things which have so much to do in making a sick room comfortable, and reducing the expenditure of labour; the latter being by no means an inconsiderable item in small households. Indeed, we believe the inventor, Mr. Stebbings, was led to it by illness in his own family, and by experiencing the difficulty in keeping up the supply of hot poultices during the small hours of the morning, when nursing seems to make exceptional claims on the watcher. The invention consists first of all of a series of perforated trays enclosed in a case, to which steam has access, the principle so far being very similar to that of vegetable steamers used in all hospital kitchens. The number of trays is, of course, limited only by the size of the case, and any reasonable number of poultices can be kept warm at one time and the cold ones re-warmed. There is thus economy of material as well as an enormous saving of time and trouble. As re-warmed poultices can hardly be used in surgical cases, the invention will be valuable chiefly for affections of the chest and neck, in which a constant supply should be kept up at all hours. It is, perhaps, hardly necessary to point out that the steam used for inhalation has no connection whatever with that used for warming the poultices, and for purposes of inhalation there is an ingenious arrangement whereby the steam can be readily medicated. The details have been carefully thought out. Safety valves

Poultice Warmer and Inhaler.

are provided, and the waste steam can be condensed by water or simply conducted to a chimney or window. The heat can be supplied either by a lamp or by gas, so that the invention is equally suitable for a cottage in the country or for a town mansion. Manifestly there will be a considerable amount of heat dissipated in the room, and it is rare that this would be objectionable during the night, at least in our English

Poultice Warmer and Inhaler.

climate. There is another important use, namely, that by it beef tea or other food can be kept ready for use at any moment. The accompanying illustrations will serve to convey a fair idea of the invention, but we shall be pleased to forward to the inventor any inquiries thought desirable by our readers. There are two sizes. One for household use and one for hospitals.

## Figures and Tables

**Figure f1:**
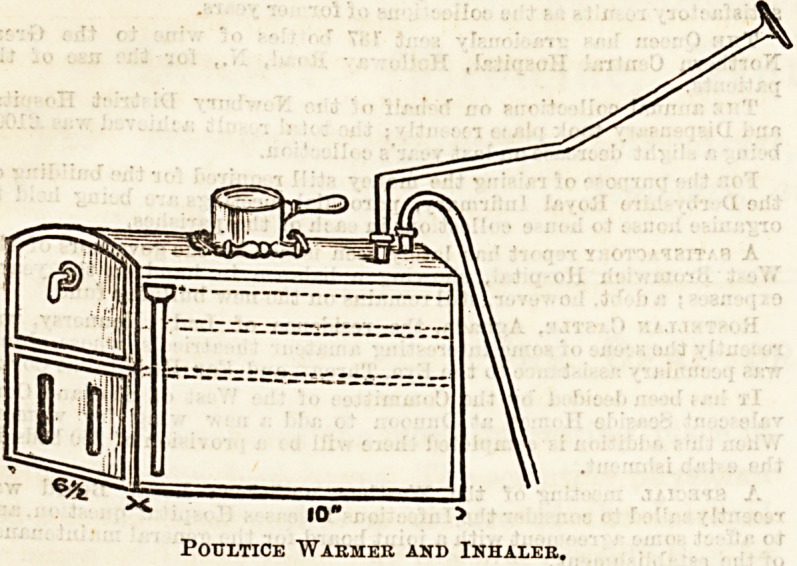


**Figure f2:**